# Label-Free Proteome Analysis of Plasma from Patients with Breast Cancer: Stage-Specific Protein Expression

**DOI:** 10.3389/fonc.2017.00014

**Published:** 2017-02-02

**Authors:** Marina Duarte Pinto Lobo, Frederico Bruno Mendes Batista Moreno, Gustavo Henrique Martins Ferreira Souza, Sara Maria Moreira Lima Verde, Renato de Azevedo Moreira, Ana Cristina de Oliveira Monteiro-Moreira

**Affiliations:** ^1^Department of Biochemistry and Molecular Biology, Federal University of Ceará (UFC), Fortaleza, Brazil; ^2^Center of Experimental Biology (Nubex), University of Fortaleza (UNIFOR), Fortaleza, Brazil; ^3^Mass Spectrometry Applications Research and Development Laboratory, Waters Corporation, São Paulo, Brazil

**Keywords:** breast cancer, mass spectrometry, MS^E^, lectin, biomarkers

## Abstract

Breast cancer is one of the most commonly diagnosed types of cancer among women. Breast cancer mortality rates remain high probably because its diagnosis is hampered by inaccurate detection methods. Since changes in protein expression as well as modifications in protein glycosylation have been frequently reported in cancer development, the aim of this work was to study the differential expression as well as modifications of glycosylation of proteins from plasma of women with breast cancer at different stages of disease (*n* = 30) compared to healthy women (*n* = 10). A proteomics approach was used that depleted albumin and IgG from plasma followed by glycoprotein enrichment using immobilized Moraceae lectin (frutalin)-affinity chromatography and data-independent label-free mass spectrometric analysis. Data are available *via* ProteomeXchange with identifier PXD003106. As result, 57,016 peptides and 4,175 proteins among all samples were identified. From this, 40 proteins present in unbound (PI—proteins that did not interact with lectin) and bound (PII—proteins that interacted with lectin) fractions were differentially expressed. High levels of apolipoprotein A-II were detected here that were elevated significantly in the early and advanced stages of the disease. Apolipoprotein C-III was detected in both fractions, and its level was increased slightly in the PI fraction of patients with early-stage breast cancer and expressed at higher levels in the PII fraction of patients with early and intermediate stages. Clusterin was present at higher levels in both fractions of patients with early and intermediate stages of breast cancer. Our findings reveal a correlation between alterations in protein glycosylation, lipid metabolism, and the progression of breast cancer.

## Introduction

Breast cancer is a clinically and biologically heterogeneous disease. The disease exhibits distinctive and complementary characteristics, which may induce tumor growth and metastatic dissemination, such as sustaining proliferative signaling, evading growth suppressors, activating invasion and metastasis, enabling replicative immortality, inducing angiogenesis, and resisting cell death (1, 2). Breast cancer represented one of the most commonly diagnosed types of cancer among women in 2015. Moreover, breast cancer is expected to account for 29% (231,840) of all new cancers of women (3).

The causes of breast cancer are unknown, and risk factors are limited primarily to females, increasing age, family history, early menarche, late menopause, pregnancy at an advanced age, and prolonged hormone replacement therapy (4). The frequent discovery of disease in the early stage allows effective treatment that facilitates a cure. Mammography, which is commonly used due to its ability to detect early forms of breast cancer, allows implementation of various treatment options. Moreover, mammography is still used for screening a population, although it is far from perfect. For example, out of 100 women with breast cancer, approximately 10% will not be diagnosed (5). Nowadays, digital breast tomosynthesis (DBT) is being proposed as an alternative for conventional mammography for breast cancer screening. It reduces and/or eliminates overlapping obscuring breast tissue. Although there are some disadvantages with DBT, this relatively lost-cost technology may be used effectively in the screening and diagnostic settings actually (6).

The increasing interest in discovering biomarkers reflects the requirement for developing highly sensitive and specific clinical tests for diagnosis and prognosis. Biomarkers can assist in the staging of several tumors, although reliable breast cancer markers are insufficient to provide precise diagnosis or classification. At least 1,000 proteins are potential biomarkers for cancer, but few are approved by the United States Food and Drug Administration (7). Some are glycoproteins, which are applied to analyzing different types of cancer (8, 9). The changes of protein expression as well as modifications such as glycosylation are frequently reported to contribute to cancer development, and these findings have stimulated research designed to develop new proteomic strategies to identify serological indicators of cancer based on different approaches to analyze protein glycosylation (10).

Because changes in protein glycosylation are associated with diseases such as cancer and immune disorders, a common strategy of glycoproteomics is using lectins as a selection tool, mainly because they bind glycoproteins present in complex mixtures. Lectins are defined as proteins of non-immune origin that are present in animals, plants, and microorganisms and bind specifically and reversibly to carbohydrates or glycoconjugates (11–13). Studies using plant lectins in biotechnology reveal the presence of highly diverse anomalous and truncated glycans in cancer cells. Further, the antitumor effects provoked by plant lectins are attributed to their ability to bind reversibly to epitopes that seem to arise during tumorigenesis (14–16).

Qualitative and quantitative analyses of plasma proteins from healthy women and women with breast cancer are presented here. We depleted plasma of albumin and IgG and then enriched for glycoproteins using *Moraceae* lectin (frutalin)-affinity chromatography and data-independent (MS^E^) label-free mass spectrometry to identify proteins that were differentially expressed as a function of disease stage. The goal of the present study was to identify differentially expressed proteins and changes in glycosylation patterns, in order to contribute the development of a protein profile that suggests an association with the development and characterization of breast cancer at different stages.

## Materials and Methods

We analyzed plasma samples from healthy women and women with one of three different stages of ductal breast cancer. The Ethics Committee of the Hospital Geral de Fortaleza approved this study (protocol number 050507/10). Blood samples were used after obtaining a patient’s written informed consent, and the study was designed and conducted in accordance with the ethical principles for medical research involving human subjects stated in the Declaration of Helsinki.

### Study Patients

A total of 40 patients had a mean age of 52.3 ± 11.7 years (case group) and 46.3 ± 14.3 years (control group). These patients were enrolled in our prospective study based on a series of 10 healthy controls and 30 patients diagnosed with ductal infiltrative carcinoma of the breast from May 2011 to June 2012 attending in the General Hospital of Fortaleza, Brazil. Women with newly diagnosed clinical and anatomopathological ductal breast cancer clinical staging (CS) I, II, and III without metastasis and other associated neoplasms, whose tumors were biopsied and diagnosed as breast cancer at the Biopsy Laboratory of the Hospital Geral de Fortaleza, were eligible for the case group (*n* = 10, each stage). Patients with breast cancer with associated malignancies, prior treatment, and a Karnofsky Index >70 (17) were excluded from the case group. The control group (*n* = 10) comprised healthy women, without a diagnosis of cancer. Women with chronic or transmitted diseases, receiving pharmacologic therapy, those with neurological or psychiatric disorders, alcoholics, smokers, or those whose data records were incomplete regarding the characteristics required for the study were excluded as well. Clinicopathological data from patients were acquired by a team of researchers at analysis of medical records and included age at diagnosis, weight, clinical and family history, menopausal status, CS, and characteristics of the tumor and showed no significant differences (Tables 1–3).

**Table 1:** **Description of the socioeconomic profile of women in study**.

Sociodemographic data	Case group (*n* = 30)	Control (*n* = 10)	χ^2^
**Marital status**			
Married	17	07	*p* = 0.006
Single	07	02	
Others^a^	06	01	
**Ethnicity**			
White	07	03	*p* = 0.12
Brown	18	06	
Others^b^	05	01	
**Education (years of study)**			
≤9 years	12	07	*p* = 0.94
10–12 years	08	03	
≥12 years	10	0	
**Family income^c^**			
≤1 MS	21	04	*p* = 0.72
2–6 MS	07	06	
≥10 MS	02	0	

*^a^Divorced, widow, and others*.

*^b^Yellow, black, and indigenous*.

*^c^Minimum salary value (MS): equivalent to $300. Difference between the groups was evaluated chi-squared test (χ^2^) with level significance of *p* < 0.05*.

**Table 2:** **Description of the clinical profile of women in study**.

Clinical profile	Case group (*n* = 30)	Control (*n* = 10)	χ^2^
Weight	67.5 ± 11.2	65.7 ± 11.1	*p* = 0.264
**Menopause status**			
Pré-menopause	14	04	*p* = 0.286
Pós-menopause	16	06	
**Nulliparity**			
Yes	05	02	*p* = 0.796
No	25	08	
**Breast-feeding**			
Yes	23	07	*p* = 0.881
No	02	01	
**Tobacco smoking**			
Yes	14	07	*p* = 0.338
Ex-smoker	11	03	
No	04	0	
**Family history**			
Yes	21	06	*p* = 0.850
No	09	04	

*Difference between the groups was evaluated chi-squared test (χ^2^) with level significance of *p* < 0.05*.

**Table 3:** **Description of the case group according to the characteristics of the tumor**.

Tumor characteristics	*n*	%
**Subtype**		
Ductal	30	100
Lobular	0	0
**Clinical staging**		
0	0	0
I	10	33.33
II	10	33.33
III	10	33.33
IV	0	0
**Size**		
Tis	0	0.0
T1 (until 2 cm)	11	36.7
T2 (2–5 cm)	7	23.3
T3 (≥5 cm)	5	16.7
T4	5	16.7
No data	2	6.7
**Lymph nodes committed**		
N−	19	63.3
N+	9	30
No data	2	6.6
**Histopathological grade**		
1	4	13.3
2	7	23.3
3	8	26.7
No data	11	36.7

### Blood Samples

Blood sample from all subjects were processed identically as follows: samples were collected by venipuncture into tubes containing a separating gel clot activator and were centrifuged at 1,300 × *g* at 4°C for 20 min. The plasma was removed, transferred in 1-ml aliquots to polypropylene tubes, and stored at −80°C.

### Isolation of an α-d-Galactose Lectin-Ligand from *Artocarpus incisa* (frutalin) Seeds

The α-d-galactose lectin-ligand frutalin was purified according to a published protocol (18). Seeds of *A. incisa* were collected in the state of Ceara, Brazil, and ground to a fine powder, delipidated in *N*-hexane, then suspended (1:10 w/v) in 0.15 M NaCl with agitation for 1 h at room temperature. The suspension was centrifuged at 10,000 × *g* for 30 min at 4°C, and the supernatant was passed through filter paper and applied to a d-galactose-agarose column equilibrated with 0.15 M NaCl. The non-retained peak was eluted with the same equilibration buffer until the absorbance at 280 nm reached 0.020. The retained peak containing frutalin was eluted with 0.2 M d-galactose in 0.15 M NaCl, dialyzed exhaustively against distilled water, and lyophilized.

### Immobilization of Frutalin on Sepharose 4B

The purified frutalin was immobilized on a Sepharose 4B resin activated with cyanogen bromide (GE Healthcare, Buckinghamshire, UK). Lectin was incubated in the presence of its inhibitor sugar (0.2 M d-galactose) with the resin preactivated in an alkaline environment. All procedures followed the instructions provided by the manufacturer (GE Healthcare).

### Immunodepletion

Albumins and IgGs were removed from plasma to enrich for less abundant proteins. Aliquots of plasma were filtered through a 0.22-mM membrane (VertipurePVDF syringe filters, Vertical), then applied to a HiTrap Albumin and IgG Depletion column (GE Healthcare) attached to an ÄKTApurifier 10 fast protein liquid chromatography (FPLC) system (GE Healthcare). Plasma (150 µl) was applied to the column, which was pre-equilibrated with a solution of 20-mM Tris–HCl, pH 7.4, 0.15 M NaCl. The non-retained material was stored at −80°C. Albumin and IgG were eluted with 0.1 M glycine–HCl buffer, pH 2.7, delivered at 1 ml/min, and absorbance was monitored at 216 and 280 nm.

### Normalization of Samples and Frutalin-Affinity Chromatography

The depleted peaks were quantified by their absorbance at 280 nm using a NanoVue Plus Spectrometer (GE Healthcare). Four pools (control, breast cancer stages I, II, and III) were formed based on the same protein concentration and were subjected to frutalin-affinity chromatography using an ÄKTApurifier 10 FPLC system (GE Healthcare). Chromatography was performed at 1 ml/min, and the absorbance of the eluate was monitored at 216 and 280 nm. All runs were performed using 1 ml of the depleted pooled plasma. The frutalin-affinity column was pre-equilibrated and washed with a solution of 20-mM Tris–HCl, pH 7.4, 0.15 M NaCl. After collecting the non-retained material (unbound-PI fraction), the retained peak (bound-PII fraction) was eluted with d-galactose in 0.2 M Tris–HCl 20 mM, pH 7 4, 0.15 M NaCl. Both fractions were dialyzed against ultrapure water and concentrated using centrifugal concentrators with molecular mass cutoff values of 5 kDa (VivaSpin, GE Healthcare).

### NanoUPLC and Label-Free Data-Independent Mass Spectrometric Analysis

The unbound-PI and bound-PII fractions from healthy women and those with breast cancer were dialyzed, concentrated, and quantified using a Nanovue Spectrometer (GE Healthcare) according to the absorbance at 280 nm. Samples were adjusted to the same protein concentration, dried, and digested with trypsin as described below. Each pooled sample (100 µg) was denatured, digested with trypsin, and analyzed using mass spectrometry. The samples were diluted in 50-mM ammonium bicarbonate, denatured in the presence of 0.2% RapiGEST SF at 80°C for 15 min in a dry bath (Waters, Milford, USA), reduced with 100-mM dithiothreitol at 60°C for 60 min, then alkylated with 300-mM iodoacetamide for 30 min in the dark at room temperature. The samples were digested with a modified trypsin (Promega) at a ratio of enzyme:protein = 1:100 (w/w) at 37°C and incubated overnight. The reaction was stopped using 10 µl of 5% trifluoroacetic acid, mixed, incubated for 90 min at 37°C, centrifuged, and the supernatant was transferred to a Waters Total Recovery vial (Waters). Tryptic peptides of yeast alcohol dehydrogenase (ADH) were added to a final concentration of 50 fmol/μl as an internal standard to estimate the amount of each sample injected into the column for absolute quantification (19). The final protein concentration was approximately 1 µg/µl.

Tryptic peptides were separated using a nanoACQUITY UPLC system (Waters) equipped with an HSS T3 C18 reverse-phase column (1.8 µm, 75 µm × 20 mm) for 110 min using a 0–40% gradient (see below) for 90 min, 40–85% for 5 min, then the column was re-equilibrated for 15 min at 35°C. The flow rate was 0.35 µl/min, and the mobile phases A and B contained 0.1% formic acid in water and 0.1% formic acid in acetonitrile, respectively. All samples were injected at least in triplicate. Data-independent analysis (MS^E^) of tryptic peptides was performed using a Synapt HDMS mass spectrometer (nanoESI-Qq-*oa*TOF) (Waters, Manchester, UK). The instrument was operated in electrospray positive-ion mode nanoESI (+) and in “V” mode with a precursor double-charge resolution ≥10,000 full width at half maximum. The mass spectrometer data were acquired using a NanoLockSpray probe channel infusion of Glu–Fib (Glu^1^) derived from fibrinopeptide B human (M + 2H)^2+^ = 785.2486, and the MS/MS Glu–Fib fragments were used for final calibration of the instrument. All analyses were performed using a Glu–Fib mass channel at 30-s intervals.

The exact mass retention times (20) of nanoLC-MS^E^ data were collected with alternative lower (3 eV) and elevated collision ramp energies (15–50 eV) applied to the argon collision cell using a scan time of 1.5 s with a 0.2-s interscan delay for each MS scan from *m/z* 50 to 2,000. The RF offset (MS profiles) was adjusted such that LC/MS data were effectively acquired from *m/z* 300 to 2,000, which ensured that any mass observed in the LC/MS^E^ data <*m/z* 300 arose from dissociations in the collision cell.

### Data Processing, Protein Identification, and Quantification

LC/MS^E^ data were processed and proteins were identified using ProteinLynxGlobalServer v.2.4 software (PLGS) with the UNIPROT (2014_09) reverse *Homo sapiens* annotated database. For searching spectra and the database, we used the default parameters of PLGS followed by a maximum of one missed trypsin-cleavage, a fixed carbamidomethyl modification and a variable oxidation modification (20, 21). The absolute quantification of each run was calculated according to the three most intense peptides (label-free Hi3 method) using ADH peptides as internal standards (21). Further, for relative quantification of identified proteins from each expression group, we selected an internal common housekeeping protein alpha-1-antichymotrypsin [UNIPROT (AACT_HUMAN)] and alpha-1-antitrypsin (A1AT_HUMAN) for normalizing expression levels, which was performed using PLGS Expression^E^ software (Waters, UK) (22). The average quantitative values of all samples were calculated, and the *p* value (*p* < 0.05) calculated using Expression^E^ software to refer to the differences between biological replicates.

## Results

**Figure 1:**
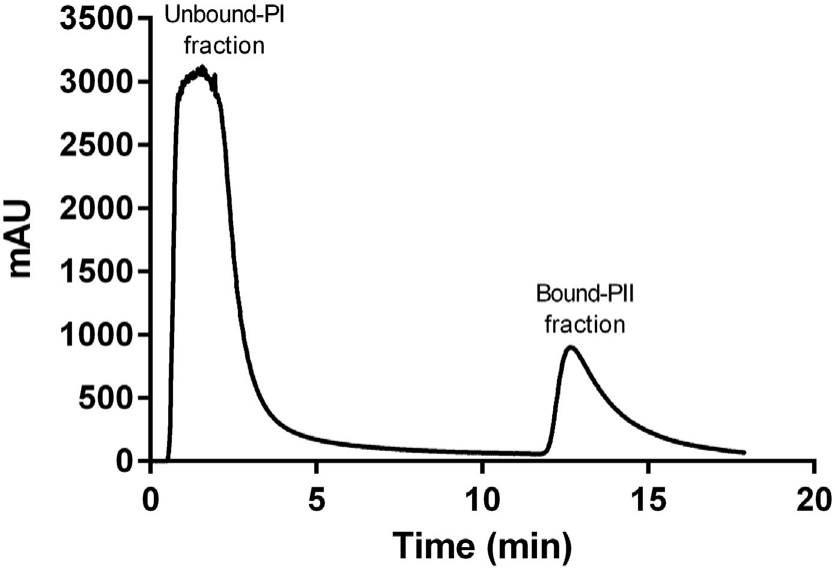
**Representative chromatogram of a depleted plasma sample prepared using affinity chromatography on an immobilized Frutalin column attached to an fast protein liquid chromatography system ÄKTApurifier 10 (GE Healthcare)**. Chromatography was performed at a constant flow of 1 ml/min and absorbance was monitored at 216 nm. The column was washed with a solution of 20 mM Tris–HCl, pH 7.4, 0.15 M NaCl. The non-retained peak (Unbound-PI fraction) was collected, and the bound material (retained peak, Bound-PII fraction) was eluted using d-galactose in a solution of 0.2 M Tris–HCl 20 mM, pH 7 4, 0.15 M NaCl.

**Figure 2:**
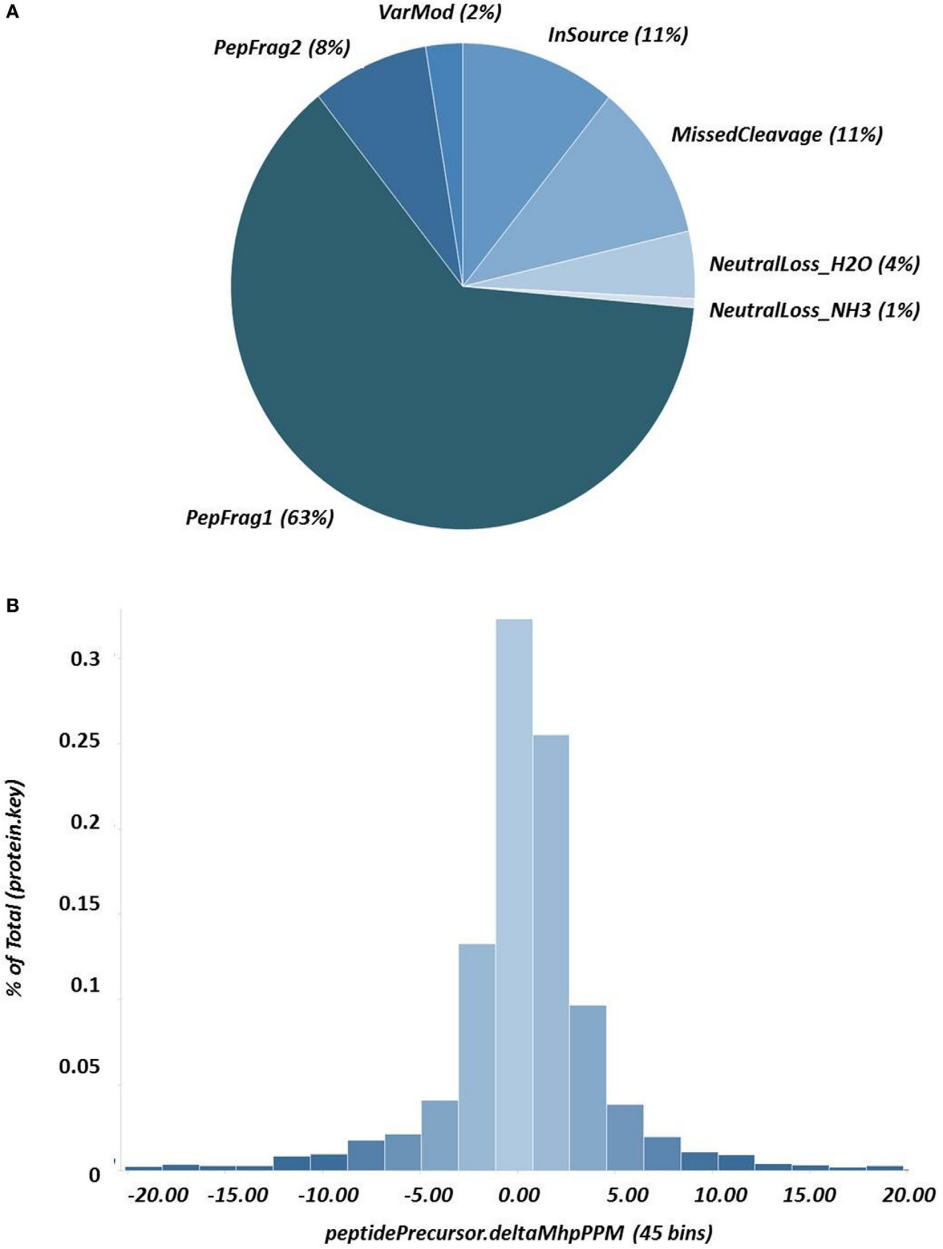
**Results of nanoUPLC HDMS^E^ analysis showing (A) the peptide-match distribution to ascertain the quality of fragmentation and digestion; and (B) the distribution of fragment masses and the exact mass accuracy for the precursor ions ± 10 ppm**.

**Figure 3:**
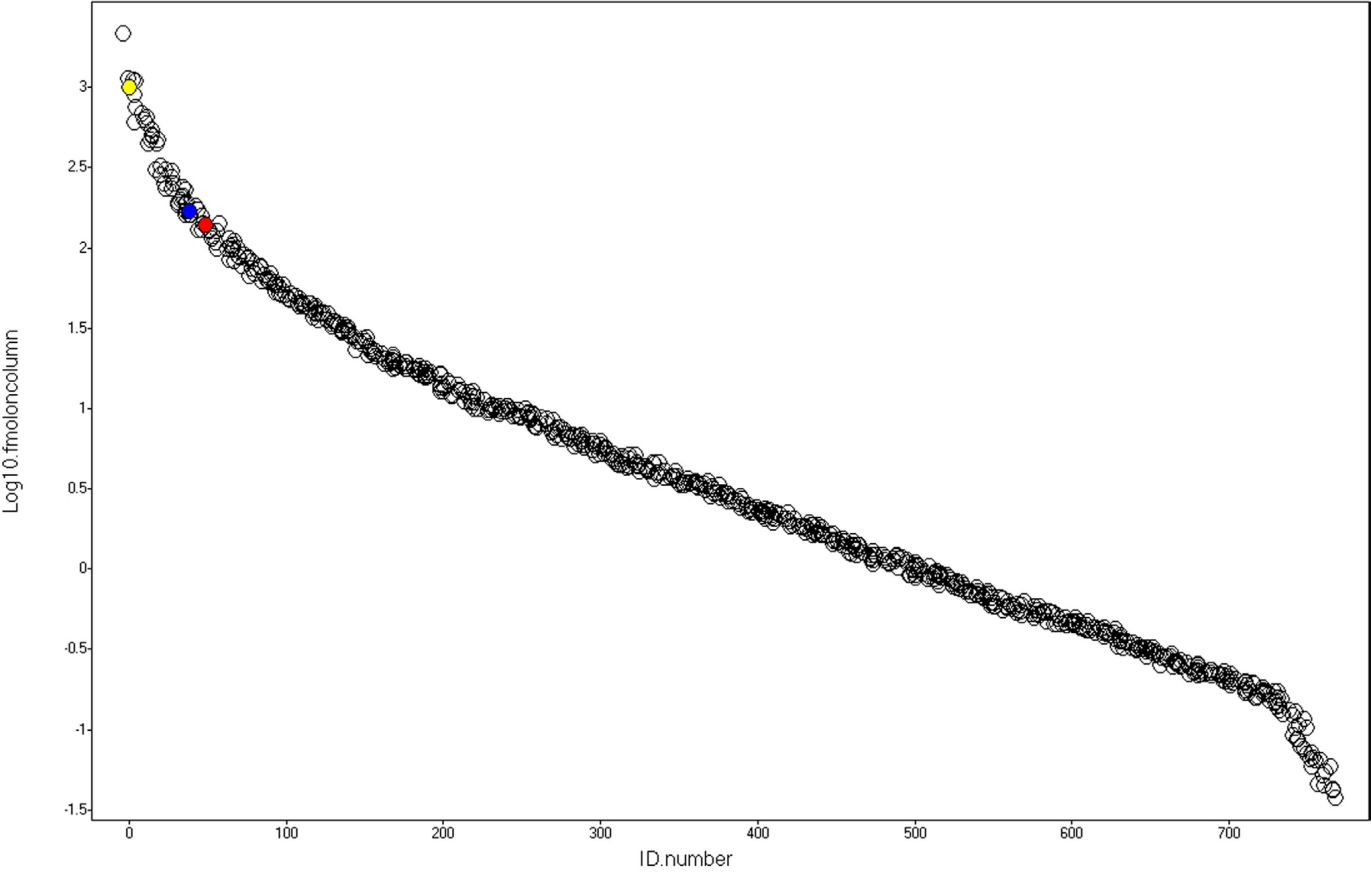
**Dynamic range**. After identification and absolute quantitation of the proteins using yeast alcohol dehydrogenase (red) as internal standard, alpha-1-antichymotrypsin (AACT_HUMAN) and alpha-1-antitrypsin (A1AT_HUMAN), represented by the blue and yellow spheres, respectively, were selected as housekeeping proteins used to normalize the data and perform label-free relative quantification of proteins present in the Unbound-PI and Bound-PII fractions, respectively.

A representative chromatogram of a depleted plasma sample prepared using affinity chromatography on an immobilized Frutalin column showing the unbound-PI and bound-PI fractions prepared from each group of subjects is illustrated in Figure 1. The analysis identified 57,016 peptides among all samples and replicates. This number of peptides corresponded to proteins that were consistently identified that corresponded to 87% of all proteins. These peptides were identified with accuracy as a normal distribution, a maximum ± 10 ppm, and a false discovery rate (FDR) = 0.3 (Figure 2A). Eleven percent of the doubly or triply charged peptides were produced by in-source fragmentation, whereas missed cleavages accounted for 11% (Figure 2B). The analysis identified 4,175 reproducible proteins with an average of 14 peptides per protein, and among them, 2,622 and 2,106 proteins were identified in the unbound-PI (FDR = 0.3%) and bound-PII (FDR = 0.2%) fractions, respectively. There was a 1,000-fold range of the concentrations of the quantified proteins (Figure 3). Qualitative analysis did not reveal proteins unique to a group’s fractions in three of three biological replicates.

After identification and absolute quantitation of the proteins of each run using yeast ADH (UNIPROT: P00330) as the internal standard, alpha-1-antichymotrypsin (UNIPROT: P01011) and alpha-1-antitrypsin (UNIPROT: P01009) were selected as housekeeping proteins to normalize expression levels and perform label-free relative quantification of proteins present in the unbound-PI and bound-PII fractions, respectively. The criterion for selecting housekeeping proteins was as follows: proteins detected in all runs under all conditions with the lowest coefficient of variance according to the quantitative counts for their respective Hi3 peptides.

Proteins were identified that were present at different levels in plasma samples from patients with stages I, II, and III breast cancer compared with those of healthy controls. Only proteins with differential expression [breast cancer:control with absolute ratios >1.5 (upregulated) and <0.66 (downregulated)] were considered. The levels of the remaining proteins with ratios between 1.5 and 0.66 were considered unchanged. The criteria stated above were met by 102 and 94 proteins in each of three biological replicates in the unbound-PI and bound-PII fractions, respectively. The breast cancer:control unbound-PI fraction comprised 14 upregulated and 1 downregulated proteins, and the bound-PII fraction comprised 10 upregulated and 15 downregulated proteins for each stage of the disease. Among these up or downregulated proteins, some of them were chosen for discussion and are shown in Tables 4 and 5.

**Table 4:** **Differentially expressed proteins identified in the plasma of patients with different stages of breast cancer compared with those of healthy subjects in the Unbound-PI fraction**.

Unbound-PI fraction				
Accession	Protein description	Score	Ratio	Expression
**Breast cancer I × control**				
K2C1_HUMAN	Keratin type II cytoskeletal 1 OS *Homo sapiens*	726.51	0.51	Down
APOC3_HUMAN	Apolipoprotein C OS *H. sapiens*	2,621.36	1.51	Up
Q86TT1_HUMAN	Full length cDNA clone CS0DD006YL02 of Neuroblastoma of *H. sapiens*	12,106.20	1.54	Up
Q5VY30_HUMAN	Plasma retinol-binding protein 1 OS *H. sapiens*	1,483.52	1.55	Up
CPN2_HUMAN	Carboxypeptidase N subunit 2 OS *H. sapiens*	145.72	1.58	Up
FIBB_HUMAN	Fibrinogen beta chain OS *H. sapiens*	33,661.36	1.58	Up
CLUS_HUMAN	Clusterin OS *H. sapiens*	12,211.18	1.63	Up
A1AG2_HUMAN	Alpha-1-acid glycoprotein 2 OS *H. sapiens*	12,778.23	1.65	Up
PON1_HUMAN	Serum paraoxonase arylesterase 1 OS *H. sapiens*	1,968.45	1.68	Up
APOA2_HUMAN	Apolipoprotein-A-II OS *H. sapiens*	4,007.37	2.46	Up
**Breast cancer II × control**				
APOA2_HUMAN	Apolipoprotein-A-II OS *H. sapiens*	4,007.37	1.51	Up
FIBB_HUMAN	Fibrinogen beta chain OS *H. sapiens*	33,661.36	1.51	Up
CLUS_HUMAN	Clusterin OS *H. sapiens*	12,211.18	1.70	Up
**Breast cancer III × control**				
FIBB_HUMAN	Fibrinogen beta chain OS *H. sapiens*	33,661.36	1.55	Up
APOA2_HUMAN	Apolipoprotein-A-II OS *H. sapiens*	4,007.37	2.27	Up

**Table 5:** **Differentially expressed proteins identified in the plasma of patients with different stages of breast cancer compared with those of healthy subjects in the Bound-PII fraction**.

Bound-PII fraction				
Accession	Protein description	Score	Ratio	Expression
**Breast cancer I × control**				
Q6N092_HUMAN	Putative uncharacterized protein DKFZp686K18196 fragment OS *Homo sapiens*	36,802.04	0.41	Down
Q9NPP6_HUMAN	Immunoglobulin heavy chain variant fragment OS *H. sapiens*	17,866.64	0.53	Down
Q8NEJ1_HUMAN	Uncharacterized protein OS *H. sapiens*	86,719.59	0.60	Down
F5GXS0_HUMAN	C4b B OS *H. sapiens*	41,296.95	0.64	Down
CLUS_HUMAN	Clusterin OS *H. sapiens*	1,846.24	1.57	Up
APOC3_HUMAN	Apolipoprotein C III OS *H. sapiens*	4,164.40	1.68	Up
Q567P1_HUMAN	IGL protein OS *H. sapiens*	85,322.29	1.92	Up
Q6GMX0_HUMAN	Uncharacterized protein OS *H. sapiens*	130,309.10	3.03	Up
**Breast cancer II × control**				
Q6N092_HUMAN	Putative uncharacterized protein DKFZp686K18196 fragment OS *H. sapiens*	36,802.04	0.27	Down
Q9NPP6_HUMAN	Immunoglobulin heavy chain variant fragment OS *H. sapiens*	17,866.64	0.52	Down
B7ZLE5_HUMAN	FN1 protein OS *H. sapiens*	14,423.90	0.55	Down
F5GXS0_HUMAN	C4b B OS *H. sapiens*	41,296.95	0.59	Down
KV116_HUMAN	Ig kappa chain V I region Roy OS *H. sapiens*	6,105.97	0.63	Down
CLUS_HUMAN	Clusterin OS *H. sapiens*	1,846.24	1.60	Up
APOC3_HUMAN	Apolipoprotein C III OS *H. sapiens*	4,164.40	1.62	Up
Q567P1_HUMAN	IGL protein OS *H. sapiens*	85,322.29	1.84	Up
Q6GMX0_HUMAN	Uncharacterized protein OS *H. sapiens*	130,309.10	2.56	Up
**Breast cancer III × control**				
Q9HCC1_HUMAN	Single chain Fv fragment OS *H. sapiens*	7,583.12	0.35	Down
Q9NPP6_HUMAN	Immunoglobulin heavy chain variant fragment OS *H. sapiens*	17,866.64	0.48	Down
Q6P089_HUMAN	IGH protein OS *H. sapiens*	16,000.74	0.49	Down
Q6N092_HUMAN	Putative uncharacterized protein DKFZp686K18196 fragment OS *H. sapiens*	36,802.04	0.50	Down
Q8NCL6_HUMAN	cDNA FLJ90170 fis clone MAMMA1000370 highly similar to Ig alpha 1 chain C region OS *H. sapiens*	36,698.85	0.58	Down
F5GXS0_HUMAN	C4b B OS *H. sapiens*	41,296.95	0.59	Down
Q6MZQ6_HUMAN	Putative uncharacterized protein DKFZp686G11190 OS *H. sapiens*	2,964.75	1.63	Up
Q6GMX0_HUMAN	Uncharacterized protein OS *H. sapiens*	130,309.10	2.64	Up

**Figure 4:**
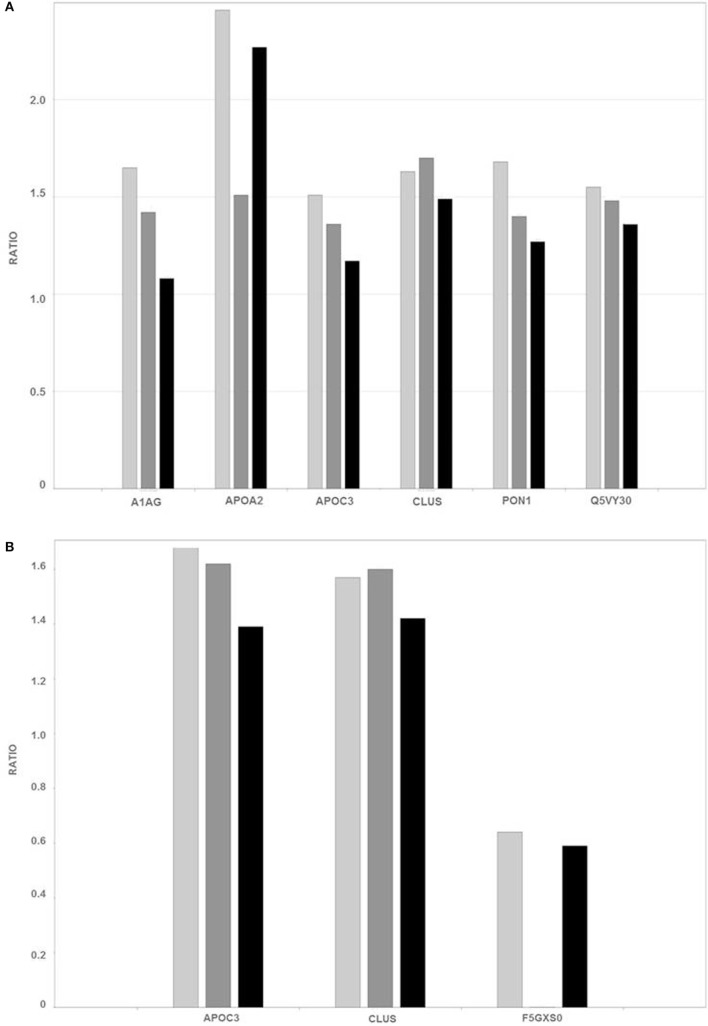
**Ratios of the levels of representative proteins [Q5VY30_HUMAN—plasma retinol-binding protein; PON1_HUMAN—serum paraoxonase arylesterase; A1AG2_HUMAN—alpha-1-acid glycoprotein; APOA2_HUMAN—apolipoprotein-A-II; CLUS_HUMAN—clusterin; APOC3_HUMAN—apolipoprotein C III; and F5GXS0_HUMAN—C4b B, detected in unbound-PI (A) and bound-PII fractions (B) of breast cancer patients and healthy controls]**. The different breast cancer stages are represented as follows: light gray (I—early), dark gray (II—intermediate), and black (III—advanced).

Figure 4 summarizes the ratios of the levels of proteins in the unbound-PI (A) and bound-PII (B) fractions from the plasma of breast cancer patients according to disease stage compared with those of healthy controls.

## Discussion

Although human plasma may represent an attractive, readily accessible source for biomarker discovery and is a more reproducible sample than serum, it presents technical challenges for MS-based proteomic analysis owing to its complexity and large dynamic range of protein concentrations (23, 24). Therefore, we consider here only the results for proteins identified in three of three biological replicates.

Tumor development is a multistep process that involves interrelated genetic, biological, and environmental factors. Tumorigenesis correlates with pathological phenotypes, clinical symptoms, and prognosis as well as proteomic changes (25). Numerous proteins were identified that are related to common biological processes such as immunity, hematopoiesis, inflammation, lipid metabolism, and cell differentiation. It is noteworthy that some proteins were excluded from analysis because of the possibility that they represent contaminants. Seven proteins were chosen for discussion, because they may be involved directly or indirectly in the development of breast cancer. Certain differentially expressed proteins identified here were identified by other investigators, which supports the contribution of these proteins to the pathogenesis of breast cancer.

### Proteomic Analysis of Unbound-PI Fractions

In the present study, we identified 2,106 proteins in the unbound-PI fractions, which corresponded to proteins or glycoproteins with no detectable frutalin-affinity under the conditions used here. Because human blood plasma is rich in glycoproteins, frutalin-affinity chromatography was essential for reducing the complexity of plasma samples and significantly improved the overall dynamic range of protein detection, enabling the identification of proteins present at very low concentrations.

Proteomics analysis revealed increased levels of plasma retinol-binding protein (Q5VY30_HUMAN) in the early stages of breast cancer and slight differences in intermediate and advanced stages. This protein binds vitamin A and is the principal and specific vitamin-A carrier in the blood. Some studies suggest that vitamin-A activity in cancer may be compromised at the level of retinol metabolism. Moreover, the interactions between retinoids and growth factors, such as transforming growth factor-β, are involved in regulating cell differentiation and proliferation (26, 27).

Oxidative stress occurs in many types of cancer cells and is associated with tumor cell proliferation (28). High levels of serum paraoxonase/arylesterase (PON1_HUMAN) were detected mainly in samples from the early stage of breast cancer. Lipid peroxidation characterizes oxidative damage to cell membranes, lipoproteins, and other lipid-containing structures. Overproduction of lipid peroxidation byproducts and alterations in the antioxidant defense system as well as serum paraoxonase and arylesterase activities are implicated in the pathogenesis of cancer (29, 30). Thus, the serum paraoxonase/arylesterase found “upregulated” in the present analysis may be a biological attempt to increase the clearance of lipid peroxidation products, thereby decreasing oxidative stress.

A high level of alpha-1-acid glycoprotein (A1AG2_HUMAN) was identified here in the early stage of breast cancer, and its level was slightly increased in the intermediate stage of breast cancer. Alpha-1-acid-glycoprotein is a plasma acute-phase glycoprotein with a high proportion of glycosylated amino acid residues. Although we found here that the levels of AAG were 1.65-fold higher in the plasma of women with breast cancer compared with those of healthy women, the mean plasma AAG increased to concentrations in breast, lung, and ovarian cancers are two-times higher compared with those of healthy people (31). Further, the concentrations of AAG increase to 5-fold greater than baseline in various acute-phase responses such as inflammation, stress, pregnancy, and myocardial infarction (32).

High levels of apolipoprotein A-II (APOA2_HUMAN) were detected here that were elevated significantly in the early and advanced stages of the disease. Lipid synthesis, which is an integrated outcome of genetic, epigenetic, and environmental factors, promotes the growth and survival of cancer cells. Certain apolipoproteins play a central role in lipid metabolism, and changes in the expression of ApoA2 occur in malignancies other than breast cancer (33, 34). Podzielinski et al. (35) suggested that lipoprotein metabolism is deregulated in ovarian cancer and that ApoA2 warrants further investigation as a marker for ovarian tumors. Moreover, there is an association between obesity and breast cancer. The tumor microenvironment, particularly that of adipose tissue, and breast cancer development seem to have an intricate relationship (36, 37).

### Proteomic Analysis of the Bound-PII Fraction

Posttranslational modifications of proteins such as glycosylation, phosphorylation, and ubiquitination play important roles in the genesis and development of cancer (25). Glycosylation is a major modification that affects charge, conformation, and stability and plays a crucial role in processes, such as cell recognition, cell–cell signaling, embryonic development, and binding of hormones and toxins (38). Changes in the glycosylation of proteins are important in many biological processes, and aberrant protein glycosylation is associated with cancer development (4). The abnormal glycosylation of proteins correlates with various diseases including cancer. Glycosylated proteins are useful targets for the development of new vaccines and are used as biomarkers for the early detection and diagnosis of disease (39).

Apolipoprotein C-III (APOC3_HUMAN) was detected in unbound-PI and bound-PII fractions. The level of ApoC3 was slightly increased only in the early stage of breast cancer compared with that in the unbound-PI fraction (1.51 breast cancer:control ratio) and was present in elevated levels in the bound-PII fractions from patients with early and intermediate stages of breast cancer. ApoC3 consists of a very low-density lipoprotein that is synthesized in the liver and intestine. A high level of ApoC3 correlates with hypertriglyceridemia and is often associated with coronary heart disease and atherosclerosis (40, 41). ApoC3 is glycosylated at threonine-74, and there are three protein glycoforms (42) that share mucin-type core-1 O-glycosylation (galactose β1−3 glycan) linked to an *N*-acetylgalactosamine residue (Galβ1−3GalNAc). Two of these three isoforms occur with a modified glycan with either one or two sialic acid residues [Galβ1−3(NeuAcα2−6)GalNAc and NeuAcα2−3Galβ1−3(NeuAcα2−6)GalNAc] (42, 43). Moreover, ApoC3 is extensively studied for potential use as a biomarker of disease, because changes in the ratio of its different glyco-isoforms occur in obesity, kidney disease, liver disease, and sepsis (44). However, there is no published information indicating an association of ApoC3 with breast cancer.

ApoC3 was differentially expressed mainly in the bound-PII fraction. ApoC3 binds reversibly to frutalin, a tetrameric lectin, which binds α-d-galactose residues and to complex carbohydrates containing Galα1-3 glycans (18, 45–47). ApoC3 is glycosylated mainly with Galβ1-3 glycans, and we show here that is was overexpressed in breast cancer patients compared with healthy controls, suggesting a change in the glycosylation pattern of ApoC3. ApoC3 is differentially glycosylated (galactose β1−3 to galactose α1−3 residues) during tumorigenesis. Nevertheless, such a possible change in glycosylation is most intriguing and supports the hypothesis that there is interplay between the changes in the glycosylation machinery and breast tumor biology.

The levels of clusterin (CLUS_HUMAN) were increased in the plasma of patients with breast cancer. In contrast to ApoC3, CLUS levels were higher in unbound-PI and bound-PII fractions at stages I and II. CLUS is an extracellular chaperone implicated in DNA repair, cell-cycle regulation, apoptotic cell death, and tumorigenesis (48). It is heavily glycosylated, and nearly 25% of its mass comprises different N-linked glycans (49), which may explain its presence in both fractions. Clusterin expression is altered in different cancers, including overexpression in human breast carcinoma (50–52), which is associated with increased tumor size, progesterone receptor-negative status, and the progression from primary to metastatic carcinoma in lymph nodes (53). The silencing of clusterin expression using small interfering RNAs decreases the invasion and migration of breast cancer cells and inhibits cell growth and metastatic progression in an orthotopic model, indicating that clusterin may contribute to the local growth-inhibitory effects in the microenvironment of the breast tumor (54).

Finally, decreased levels of C4b_B (F5GXS0_HUMAN) were detected in bound-PII fractions in all stages of breast cancer. Although some studies discuss the relationship of the inflammatory process in the immune microenvironment of tumors, few reports establish the influence of the protein components of the complement cascade in tumorigenesis. The complement system plays an important role in innate immunity and immunity to tumors. Rutkowski et al. (55) concluded that complement proteins may promote carcinogenesis; facilitate the deregulation of mitogenic signaling pathways; stimulate angiogenesis; induce resistance to apoptosis; sustain cellular proliferation, invasion, and metastasis; and allow tumor cells to escape from immunosurveillance. These possibilities raise a new view of the role of the complement system in cancer.

## Conclusion

In summary, our study employed a proteomic approach that included a frutalin lectin-affinity chromatography and data-independent (MS^E^) label-free mass spectrometry analysis that could serve as experimental design for the initial stage of studies in breast cancer proteomics research. Additionally we used an easily obtained material, such as plasma, rather than clinical samples such as tissue, often difficult to obtain.

We show here that proteins from unbound and bound fractions using lectin-affinity chromatography were present at different levels in plasma from patients with breast cancer at three different stages compared with those of healthy control subjects. The proteins plasma retinol-binding protein, serum paraoxonase/arylesterase, alpha-1-acid glycoprotein, apolipoprotein A-II, apolipoprotein C-III, clusterin, and C4b_B were differentially expressed. Interestingly, a few of the proteins that we identified were also observed in other studies, suggesting that this general approach is reproducible. However, until now there is no published information indicating an association of apolipoprotein C-III with breast cancer. Since the disease might be associated with a change in the amount of a protein but also in the structure of a given posttranslational modification, such as glycosylation, our strategy represents a potentially valuable approach for the discovery of new protein targets in breast cancer.

Accordingly, our method yielded a list of proteins for subsequent investigation. Finally, the differential expression data presented here contribute to the development of a profile or a panel of certain proteins that suggest an association with the development and characterization of breast cancer at different stages.

## Ethical Standards

All procedures performed in studies involving human participants were in accordance with the ethical standards of the institutional and/or national research committee and with the 1964 Helsinki declaration and its later amendments or comparable ethical standards.

## Author Contributions

Study conception and design: AM-M and ML. Acquisition of data: ML and FM. Analysis and interpretation of data: ML, FM, and AM-M. Drafting of manuscript: ML and FM. Critical revision: AM-M, RM, FM, GS, and SV.

## Conflict of Interest Statement

The authors declare that the research was conducted in the absence of any commercial or financial relationships that could be construed as a potential conflict of interest. The reviewer BK and handling Editor declared their shared affiliation, and the handling Editor states that the process nevertheless met the standards of a fair and objective review.
